# P02.67. An alternative for Chronic Fatigue Syndrome, an observational case series

**DOI:** 10.1186/1472-6882-12-S1-P123

**Published:** 2012-06-12

**Authors:** H Woodfield, M Dickholtz, B Bell, L Jacobs

**Affiliations:** 1 Upper Cervical Research Association, Bellingham, USA; 2 National Upper Cervical Chiropractic Association, Monroe, USA; 3 Advocate Good Shepherd Hospital, Barrington, USA

## Purpose

Chronic Fatigue Syndrome (CFS) has an elusive diagnosis and etiology. Treatment focuses on alleviation of symptoms and improving a patient’s quality of life. The primary objective was to observe and record changes in a subject’s health related quality of life (HRQoL), using the SF 36-Item Health Survey (SF-36), before and six months after a National Upper Cervical Chiropractic Association (NUCCA) atlas correction. The Pittsburgh Sleep Quality Index (PSQI) was used to observe changes in a subject's sleep quality.

## Methods

Nineteen subjects diagnosed as having CFS according to the 1994 Centers for Disease Control and Prevention (CDC) diagnostic criteria were studied. Patients who were fatigued six (6) or more months and who met four (4) or more diagnostic criteria were psychiatrically evaluated, and then underwent lab testing and SPECT imaging. Data collection and study administration were conducted using a practice-based research-based protocol. Patients were monitored for a period of six months to insure Atlas alignment was maintained and then retested.

## Results

SF-36 results at the end of the study, by a paired t-test of SF-36 data (n=19) revealed a significant increase in the General Health component, from 30.3 pre to 60.9 post (p<0.03), and in the Mental Health component, from 68.6 to 74.7 (p<0.02). The overall PSQI score significantly decreased from 12.1 to 6.1 (p<0.05). SPECT scans and lab testing were inconclusive.

## Conclusion

If correction of atlas misalignment in clinically diagnosed CFS patients is the single variable that appears responsible for self-reported improvement of functional and mental health status, then further study is warranted to determine the utility of this intervention in patient care. The study was limited by the lack of a control group and that care was provided by only one practitioner.

